# Noble element coatings on endotracheal tubes for ventilator-associated pneumonia prevention: A systematic review and meta-analysis of randomized controlled trials in emergency care settings

**DOI:** 10.1097/MD.0000000000039750

**Published:** 2024-09-20

**Authors:** Nabeel Ashiq, Fouzia Munir, Safeer Khan, Adil Yousaf, Malik Hassan Mahmood

**Affiliations:** a Department of Emergency Medicine, Hamad Medical Corporation, Doha, Qatar; b Department of Pharmaceutical Sciences, Institute of Chemical Sciences, Government College University, Lahore, Pakistan; c Green Health Pharmaceutical Company, Riyadh, Kingdom of Saudi Arabia; d Department of Pharmacology, Faculty of Pharmaceutical Sciences, Government College University, Faisalabad, Pakistan.

**Keywords:** emergency care settings, intensive care units, noble element–coated endotracheal tubes, silver-coated endotracheal tubes, ventilator-associated pneumonia

## Abstract

**Background:**

Ventilator-associated pneumonia (VAP) is the second most prevalent nosocomial infection in emergency care settings. An emerging strategy to reduce this risk involves coating endotracheal tubes (ETTs) with noble elements, leveraging the antimicrobial properties of elements such as silver, gold, and palladium. This systematic review and meta-analysis aimed to evaluate the effectiveness of noble element coatings on ETTs in reducing VAP incidence rates, mortality, duration of mechanical ventilation, and length of stay in the intensive care unit (ICU).

**Methods:**

Adhering to the Preferred Reporting Items for Systematic Reviews and Meta-Analyses guidelines, a comprehensive search was conducted across 5 databases up to 2024. The quality of the randomized controlled trials was assessed using the updated Cochrane Risk of Bias (RoB) 2 tool. A random-effects meta-analysis was performed using RevMan 5.4 Comprehensive Meta-Analysis software. Statistical heterogeneity among the studies was evaluated using the Higgins I^2^ value, with *P* < .05 indicating statistical significance.

**Results:**

Seven randomized controlled trials from 5 countries were identified. Four studies had some concerns regarding bias, 2 had a high RoB, and 1 had a low RoB. Noble metal–coated ETTs resulted in a lower incidence of VAP compared to noncoated ETTs (relative risk, 0.76 [95% confidence interval [CI], 0.60–0.96]). However, there was no significant difference in mortality rates (relative risk, 1.06 [95% CI, 0.93–1.20]), duration of mechanical ventilation (mean difference, −0.10 [95% CI, −1.62 to 1.41]), and ICU stay (mean difference, 0.07 [95% CI, −1.98 to 2.12]).

**Conclusion:**

Noble metal–coated ETTs effectively reduce the incidence of VAP but do not significantly impact mortality rates, the duration of mechanical ventilation, or ICU stay. Therefore, these coated ETTs should be integrated into a holistic care plan addressing all aspects of patient management in emergency care settings.

## 1. Introduction

Ventilator-associated pneumonia (VAP) is the second most prevalent nosocomial infection among patients in intensive care units (ICUs).^[1,2]^ This condition is associated with increased morbidity and mortality rates, higher healthcare costs, and prolonged hospital stays.^[1]^ Studies report that VAP affects 5% to 40% of patients who undergo invasive mechanical ventilation for >2 days. The attributable mortality rate for VAP is estimated to be ≈10%.^[3]^ Furthermore, it imposes significant economic burdens, with treatment costs per patient ranging from £6000 to £22,000 in the United Kingdom^[4]^ and from $25,000 to $28,000 in the United States.^[5]^

The prevention of VAP within ICUs encompasses a diverse range of preventive strategies.^[6,7]^ These strategies are underpinned by evidence-based practices including, but not limited to, antiseptic oral hygiene, continuous elimination of bacteria-laden subglottic secretions, and the special design of endotracheal tubes (ETTs).^[8]^ ETT, a critical component for mechanical ventilation, significantly contributes to the risk of VAP by providing an environment for bacterial colonization and subsequent biofilm development.^[9]^ An emerging strategy to reduce this risk involves coating ETTs with antiseptic and antimicrobial substances, such as antibiotics, chlorhexidine, and noble elements.^[10]^ ETTs coated with noble elements leverage the antimicrobial properties of elements such as silver, gold, and palladium.^[11]^

In the literature, the coating of noble elements on ETTs has been demonstrated to effectively prevent the incidence of VAP. A systematic review and meta-analysis conducted by Li et al^[12]^ analyzed 2 randomized controlled trials (RCTs) involving 1630 participants and concluded that silver-coated ETTs significantly reduced the incidence of VAP. Specifically, compared to noncoated ETTs, the silver-coated ETTs were associated with a lower incidence of VAP (relative risk [RR], 0.64 [95% confidence interval [CI], 0.43–0.96]) and fewer device-related adverse events (RR, 0.53 [95% CI, 0.32–0.88]). However, there was no statistically significant difference observed in total mortality (RR, 1.14 [95% CI, 0.99–1.30]).^[12]^ Similarly, the Cochrane review by Tokmaji et al^[13]^ supported these findings, which included 3 RCTs with 2081 participants. It was concluded that silver-coated ETTs reduced the risk of VAP from 6.7% to 3.5%, particularly within the first 10 days of intubation for patients requiring mechanical ventilation for >24 hours.^[13]^

Despite their contributions, these reviews focused exclusively on silver-coated ETTs and concentrated solely on the outcome of VAP incidence. In addition, these reviews collectively analyzed only 3 studies, as one was common among the reviews and another was an unpublished clinical trial. This identifies a significant gap in the literature, underscoring the need for a comprehensive systematic review and meta-analysis on ETTs coated with noble elements, including silver, gold, and palladium. In addition, there is a need to evaluate the effects of these noble element–coated ETTs on multiple outcomes, including the incidence of VAP. Therefore, the objective of this systematic review and meta-analysis was to evaluate the effectiveness of noble element coatings on ETTs in preventing VAP in emergency care settings. This study aims to synthesize existing evidence on the antibacterial properties of these coatings and their impact on VAP incidence rates, mortality, duration of mechanical ventilation, and length of ICU stay.

## 2. Methods

### 2.1. Search strategy

Following the guidelines of the Preferred Reporting Items for Systematic Reviews and Meta-Analyses,^[14]^ we conducted a thorough search across 5 databases, including PubMed, Scopus, Cochrane Library, ScienceDirect, and Google Scholar, to find peer-reviewed literature in full text from the start until March 2024. In addition, relevant studies were found by conducting a bibliographic analysis of relevant systematic reviews and meta-analyses.

The search was performed with specific keywords including “noble element coatings,” “endotracheal tubes,” and “ventilator-associated pneumonia.” After removing duplicates, the titles, abstracts, and full-text publications were thoroughly evaluated to eliminate research that did not meet the inclusion criteria. The search strategies utilized for each database and the number of retrieved results are shown in Supplementary Data 01, Supplemental Digital Content, http://links.lww.com/MD/N600.

### 2.2. Selection of studies

The inclusion criteria consisted of RCTs in the English language only, with 2 intervention arms. There were no limitations on the criteria used to diagnose pneumonia or assess its incidence.

The criteria for inclusion in this systematic review and meta-analysis were designed utilizing the Population, Intervention, Comparator, Outcome framework. We encompassed a broad population of intubated patients undergoing mechanical ventilation without limitations regarding age, gender, ethnicity, or existing comorbidities. The intervention of interest was the application of ETTs coated with noble elements. These were compared against noncoated or conventional ETTs. Finally, the primary outcome was the incidence rate of VAP, while secondary outcomes included mortality rate, the duration of mechanical ventilation, and the duration of ICU stay.

Three authors (NA, FM, and SK) carried out an in-depth assessment of the selected full-text studies and resolved any eligibility concerns through discussion.

### 2.3. Quality appraisal and data extraction of included studies

Each author independently evaluated the steps of data extraction and quality assessment. During the data extraction phase, a standardized data extraction sheet was employed. Extracted data from each study encompassed study details, study design, sample demographics, concomitant pneumonia prevention interventions, ETT type, VAP definition, number of VAP episodes, mortality rate, duration of mechanical ventilation, length of stay, conclusions, and limitations. In cases of discrepancies or disagreements in data extraction, the authors engaged in a discussion to arrive at a unanimous agreement.

The included studies were evaluated for their quality and Risk of Bias (RoB) using the updated Cochrane RoB tool,^[15]^ also known as the RoB 2.0 tool. The Cochrane RoB tool comprises criteria that evaluate different aspects of research design and implementation. The 5 areas encompass bias stemming from the randomization technique, bias originating from variations from planned interventions, bias resulting from incomplete outcome data, bias in the measurement of the final outcome, and bias in the selection of the reported findings. A decision was made for each domain to determine whether the RoB was low, high, or considerable concern. Each study was evaluated based on the following criteria to determine an overall judgment.

1. Low RoB: The study demonstrates low bias in all 5 dimensions.
2. Some concerns: The study has some concerns in at least 1 area but does not present a high RoB in any area.
3. High RoB: The study exhibits a high RoB in at least 1 area, or it raises concerns in >1 area.

### 2.4. Statistical analysis

A random-effects meta-analysis was conducted utilizing Revman 5.4 Comprehensive Meta-Analysis software. Statistical heterogeneity among the studies was evaluated using the Higgins I^2^ value, with thresholds set at I^2^ ≤ 25% indicating low heterogeneity, 25%–50% for moderate, and ≥50% for high heterogeneity.^[16]^ *P* < .05 was deemed to indicate statistical significance. Due to the limited number of studies (<10), a funnel plot analysis was not conducted.

## 3. Results

### 3.1. Literature search

**Figure 1.**
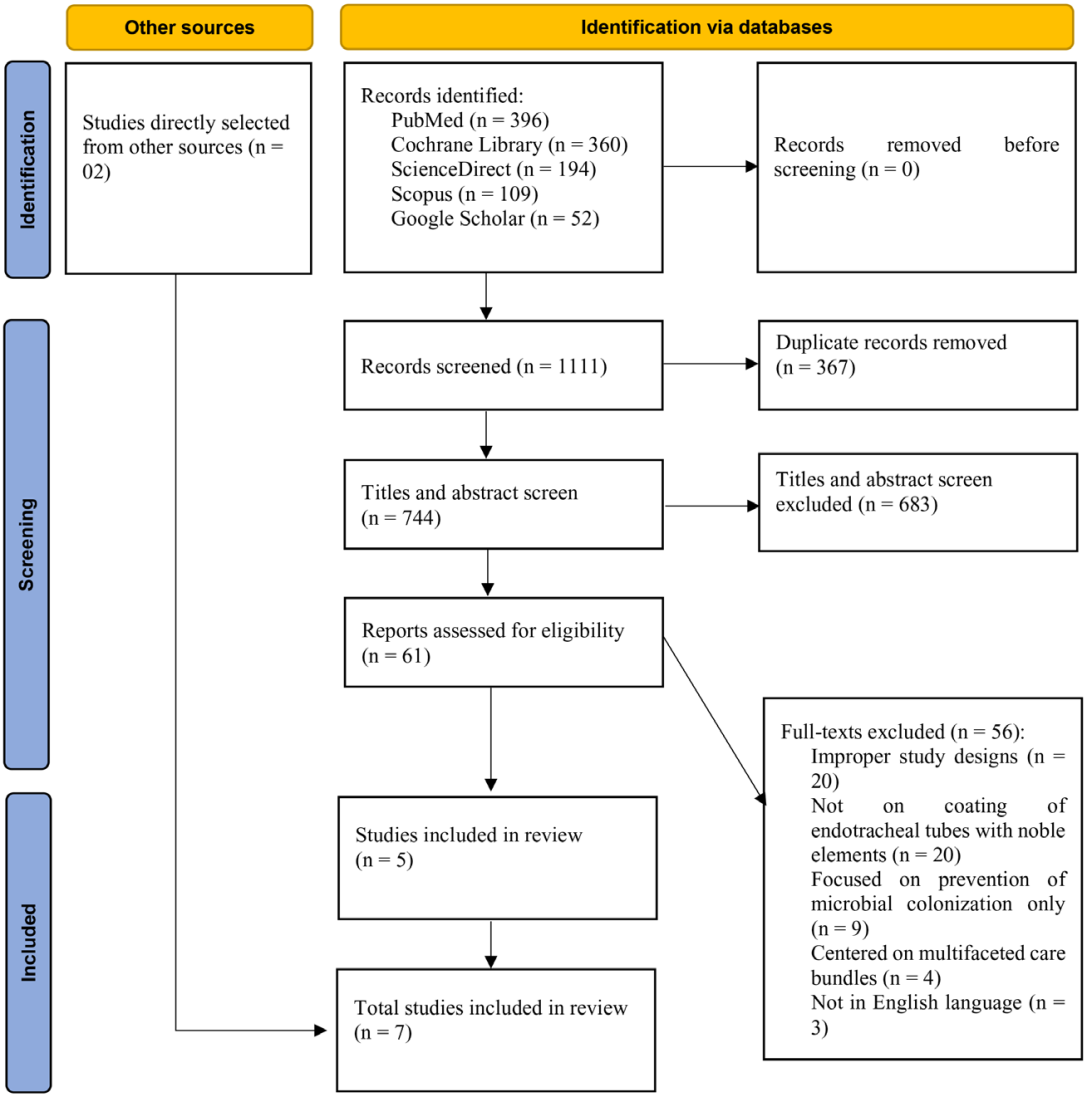
Preferred Reporting Items for Systematic Reviews and Meta-Analyses flowchart of the searching and screening studies.

A comprehensive search of 5 databases identified a total of 1111 studies. After the removal of 367 duplicate records, 744 studies remained for title and abstract screening. Subsequently, 683 studies were excluded based on predefined exclusion criteria. This resulted in 61 full-text studies being assessed for eligibility. Of these, 56 studies were excluded for the following reasons: 20 had improper study designs, 20 did not investigate the coating of ETTs with noble elements, 9 focused exclusively on the prevention of microbial colonization, 4 centered on multifaceted care bundles, and 3 were not in English. Ultimately, 5 articles met the inclusion criteria and were selected for the final analysis.^[17–21]^ In addition, 2 studies were directly sourced,^[22,23]^ resulting in a total of 7 selected studies. Figure 1 illustrates the process of searching and screening the studies.

### 3.2. Quality assessment of included studies

Based on the assessment criteria of the updated RoB 2 tool, the majority of the RCTs (n = 4; 57%) in the systematic review were categorized as having some concerns regarding the RoB. This was followed by 2 studies that were identified as having a high RoB. In contrast, only 1 study received a rating of low RoB. The primary reason for the conclusion of the RoB, which was a concern in the studies, was the domain of bias in outcome assessment. Similarly, the high RoB in 2 studies was attributed to noncompliance with the domain of bias arising from deviations from intended interventions. The quality assessment of included studies is presented in Table 1.

**Table 1:** Assessment of the quality of included studies utilizing the updated Cochrane Risk of Bias 2 tool.^[15]^

Criterion	Tincu et al^[17]^	Damas et al^[18]^	Mahmodiyeh et al^[19]^	Zampieri et al^[20]^	Mahmoodpoor et al^[22]^	Afessa^[23]^	Kollef et al^[21]^
D1	+	+	+	+	+	+	+
D2	?	+	−	?	−	+	+
D3	+	+	+	+	+	+	+
D4	+	+	?	+	?	?	?
D5	+	+	+	+	+	+	+
D	Some concern	Low	High	Some concerns	High	Some concerns	Some concerns

A response of “Low” was indicated by a “+,” and a response of “High” was indicated by a “−,” while a response of “some concerns” was indicated by “?.” D1 for bias arising from the randomization process, D2 for bias due to deviations from intended interventions, D3 for bias due to missing outcome data, D4 for bias in the measurement of the outcome, D5 for bias in the selection of the reported result, and D for overall risk of bias.

### 3.3. Baseline characteristics of included studies

Among the 7 RCTs, 2 studies were conducted in Iran and North America, followed by 1 study in Romania, Belgium, and Brazil. The total sample size across these studies was 2418 participants, with an average age of 56 years; 61% of the participants were male.

The primary reason for ventilation in the majority of cases was due to medical causes. Previous antibiotic use was reported in 68.8% of the participants in the intervention group and 75.4% in the control group according to data from 2 studies.^[18,20]^ In addition, the mean Acute Physiology and Chronic Health Evaluation II score reported in 3 studies was 20.6 for the intervention group and 21 for the control group.^[21–23]^

Regarding comorbidities, 17.6% of participants in the intervention group and 19% in the control group had coexisting medical conditions, as reported in 3 studies. Finally, the diagnostic criteria varied across all the included studies. A summary of the baseline characteristics of the included studies is presented in Table 2.

**Table 2:** Summary of baseline characteristics in included studies.

Study	Country	Study design	Sample demographics	Reason for ventilation	Previous antibiotic use	Mean APACHE II score	Comorbidities	Diagnostic criteria for VAP
Tincu et al^[17]^	Romania	RCT	n = 188; mean age, 39.5 years; 64.5% male	47.4% respiratory failure, 19.3% CV failure, 33% CNS disorders	NR	NR	Intervention: 13% Control: 14%	CDC diagnostic criteria 2021
Damas et al^[18]^	Belgium	Multicenter, randomized, controlled, double-blind, prospective study	n = 323; median age, 67.6 years; 55.5% male	33% respiratory, 19.7% neuro, 16.8% cardiac, and 30.5% other cases	Intervention: 70.8% Control: 78.1%	NR	Median Charlson score of 2 for both intervention and control	New infiltrate on chest X-ray + fever (>38.3 °C) or hypothermia (<36 °C), leukocytosis (>11.10^9^ WBC/L) or leukopenia (<4.10^9^ WBC/L), plus at least one of purulent secretions, FiO_2_ increase ≥0.2 or PEEP increase ≥3-cm H_2_O for 2 days, and culture >10^6^ CFU/mL from endotracheal specimen or >10^4^ CFU/mL from BAL, after 48 h of ventilation.
Mahmodiyeh et al^[19]^	Iran	Single-blind clinical trial study	n = 108; mean age, 45.4 years	NR	NR	NR	Intervention: 19.6% Control: 20.3%	Fever, purulent secretion, leukocytosis, and decreased oxygen saturation.
Zampieri et al^[20]^	Brazil	Pilot single-blinded RCT	n = 108; mean age, 57 years; 54.1% male	72.7% medical and 27.3% surgical cases	Intervention: 66.7% Control: 72.7%	NR	Intervention: 20.3% Control: 22.9%	New/progressive radiographic consolidation or infiltrate, plus at least 2 of Temp >38°C, WBC ≥12,000/mm³ or <4000/mm³, and purulent secretions.
Mahmoodpoor et al^[22]^	Iran	RCT	n = 90; mean age, 60 years; 67% male	51.2% medical, 27.3% surgical, and 21.1% trauma cases	NR	Intervention: 22 (19–26) Control: 21 (20–24)	NR	Patients on mechanical ventilation >48 h and by using clinical pulmonary infection score.^[24]^
Afessa^[23]^	North America	Retrospective cohort analysis of 1 previous trial^[21]^	n = 93; mean age, 61.6 years; 66.4% male	NR	NR	Intervention: 18.4 ± 5.9 Control: 20.3 ± 7.0	NR	Micro-organisms present at a concentration of ≥10^4^ CFU/mL in BAL fluid
Kollef et al^[21]^	North America	Prospective, randomized, single-blind, controlled study	n = 1508; mean age, 61.5 years; 58% male	18.9% medical, 30.2% surgical, 34.9% trauma cases, 16% other cases	NR	Intervention: 21.5 (7.5) Coated: 21.6 (7.5)	NR	BAL fluid culture ≥10^4^ CFU/mL in patients intubated ≥24 h

APACHE = Acute Physiology and Chronic Health Evaluation, BAL = bronchoalveolar lavage, CDC = Centers for Disease Control and Prevention, CNS = central nervous system, CV = cardiovascular, FiO_2_ = fraction of inspired oxygen, NR = not reported, PEEP = positive end-expiratory pressure, RCT = randomized controlled trial, VAP = ventilator-associated pneumonia, WBC = white blood cell count.

### 3.4. Summary of findings from included studies

While all included studies used standard noncoated ETTs as a control group, the intervention groups were divided into those with silver-coated ETTs and those with noble metal alloy (silver, gold, and palladium) ETTs.

There were 4 studies that focused solely on silver-coated ETTs. Regarding the incidence of VAP, Kollef et al^[21]^ found that silver-coated ETTs effectively lowered VAP incidence compared to standard ETTs. Similarly, Mahmodiyeh et al^[25]^ reported significant reductions in VAP incidence among patients using silver-coated ETTs. However, 1 study did not support these findings. Mahmoodpoor et al^[22]^ compared Bactiguard (silver-coated) and Taperguard (subglottic suctioning) ETTs, showing no difference in preventing VAP and ICU mortality, as well as similar durations of mechanical ventilation and ICU stay. Consistent with these secondary outcomes, Kollef et al^[21]^ also found no significant differences in mortality rates, duration of mechanical ventilation, and ICU stay between groups. Afessa et al^[23]^ focused only on mortality rates and found that silver-coated ETTs reduced mortality compared to standard noncoated ETTs.

Three studies compared noble metal alloy ETTs to standard noncoated ETTs. Tincu et al^[17]^ concluded that this alloy significantly reduced VAP incidence, mechanical ventilation duration, and ICU stay although no significant differences were observed in mortality rates. Similarly, Damas et al^[18]^ reported a notable decrease in VAP incidence and mechanical ventilation duration for noble metal alloy ETT but found no difference in ICU stay or mortality rates. Finally, Zampieri et al^[20]^ explored the efficacy of gold, silver, and palladium alloy coatings on various devices, including ETTs. However, they found no significant difference between the groups. The summary of the intervention characteristics of the included studies is presented in Table 3.

**Table 3:** Summary characteristics of intervention in included studies.

Study	Experimental						Control					
	Intervention type	Sample size	VAP incidences, %	Mortality rate, %	Duration of ventilation, d	ICU stay, d	Intervention type	Sample size	VAP incidences, %	Mortality rate, %	Duration of ventilation, d	ICU stay, d
Tincu et al^[17]^	Noble metal alloy ETT	97	27.83	2.7	3.2	7.11	Standard noncoated ETT	83	43.16	4.3	5.03	10.14
Damas et al^[18]^	Noble metal alloy ETT	168	6.5	41.1	5.5	11	Standard noncoated ETT	155	11.6	41.9	6	11
Mahmodiyeh et al^[19]^	Silver-coated ETT	54	18	NR	NR	NR	Standard noncoated ETT	54	18	NR	NR	NR
Zampieri et al^[20]^	Noble metal alloy ETT	48	12.5	35.4	NR	9.75	Standard noncoated ETT	55	7.3	40	NR	9.4
Mahmoodpoor et al^[22]^	Silver-coated ETT	45	31	17.8	17.8	21.2	Subglottic ETT	45	20	8.9	15.9	17.8
Afessa^[23]^	Silver-coated ETT	37	NR	14	NR	NR	Standard noncoated ETT	56	NR	36	NR	NR
Kollef et al^[21]^	Silver-coated ETT	766	4.8	30.4	3.2	7	Standard noncoated ETT	743	7.5	26.6	3.2	7
Mean (SD)		173.5	16.7 (10.9)	23.5 (14.5)	7.4 (7)	11.2 (5.8)		170	17.9 (13.4)	26.2 (16.2)	7.5 (5.7)	11 (4)

ETT = endotracheal tube, ICU = intensive care unit, NR = not reported, SD = standard deviation, VAP = ventilator-associated pneumonia.

### 3.5. Meta-analysis

In the meta-analysis, 4 outcomes were examined regarding the impact of noble element coatings on ETT.

#### 3.5.1. Incidence of VAP

**Figure 2.**
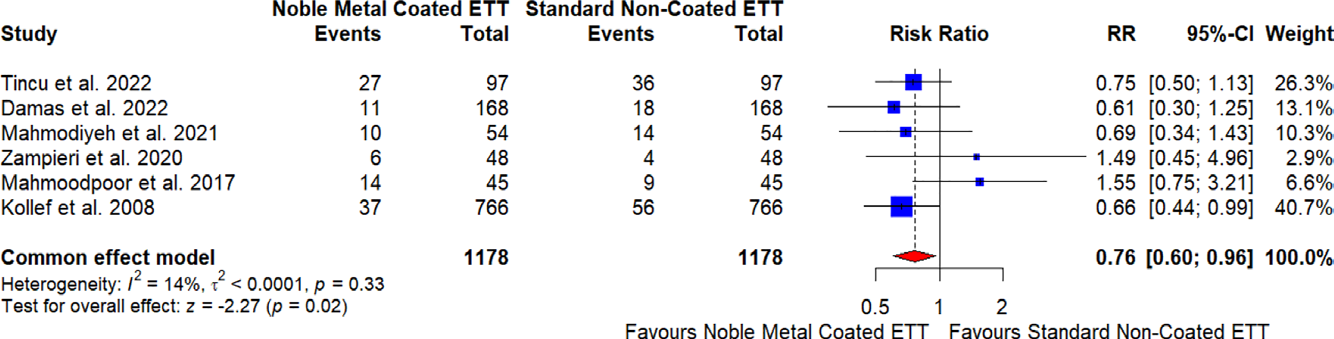
Incidence of ventilator-associated pneumonia for noble element coated endotracheal tube (ETT) versus standard noncoated ETT. CI = confidence interval, RR = relative risk.

The meta-analysis of 6 studies^[17,18,20–22,25]^ investigating the effect of noble element–coated ETTs on VAP showed that these coated tubes reduced the incidence of VAP. The overall risk ratio (RR) was 0.76, indicating a 24% reduction in risk compared to standard noncoated ETTs, with a 95% CI of 0.60 to 0.96 and a statistically significant result (*P* = .02). The heterogeneity among studies was low (I^2^ = 14%), suggesting low heterogeneity across these studies (Fig. 2).

The sensitivity analysis revealed that the overall effect size was somewhat sensitive to the exclusion of specific studies, particularly Kollef et al^[21]^ and Damas et al,^[18]^ with the RR ranging from 0.82 to 1.09, and moderate heterogeneity (I^2^ ranging from 42% to 71%; Supplementary Data 02, Supplemental Digital Content, http://links.lww.com/MD/N601). Egger test for publication bias showed no significant bias (*P* = .8870).

#### 3.5.2. Mortality rate

**Figure 3.**
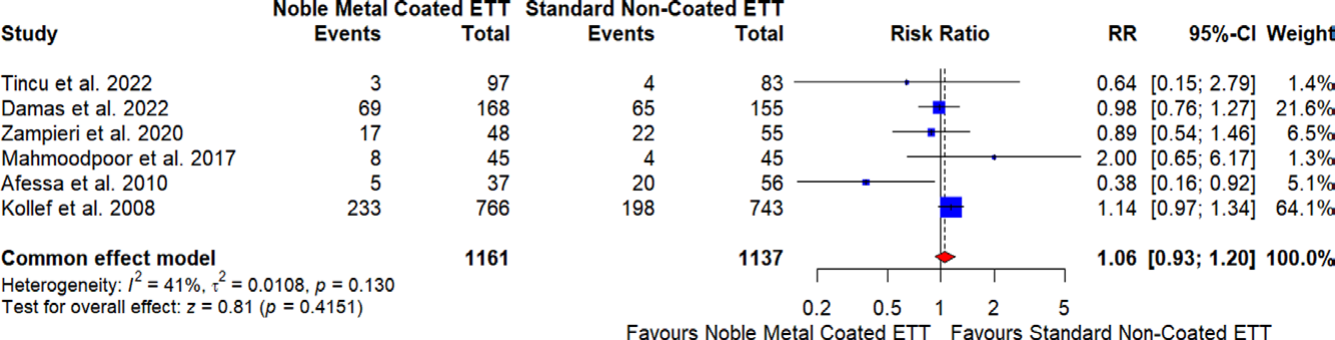
Mortality rate for noble element coated endotracheal tube (ETT) versus standard noncoated ETT. CI = confidence interval, RR = relative risk.

The meta-analysis of 5 studies^[17,18,20–23]^ investigating the effect of noble element–coated ETTs on mortality rates showed that these coated tubes did not significantly reduce mortality compared to standard noncoated ETTs. The overall RR was 1.06, indicating no significant difference in mortality risk, with a 95% CI of 0.93 to 1.20 and a nonsignificant result (*P* = .4151). The heterogeneity among studies was moderate (I^2^ = 41%), indicating some variability in the results (Fig. 3).

The sensitivity analysis revealed that the overall effect size was relatively stable across different study exclusions, with RR ranging from 1.04 to 1.09 and moderate heterogeneity (I^2^ ranging from 35% to 50%; Supplementary Data 02, Supplemental Digital Content, http://links.lww.com/MD/N601). The Egger test for publication bias showed no significant bias (*P* = .3217).

#### 3.5.3. Duration of mechanical ventilation

**Figure 4.**
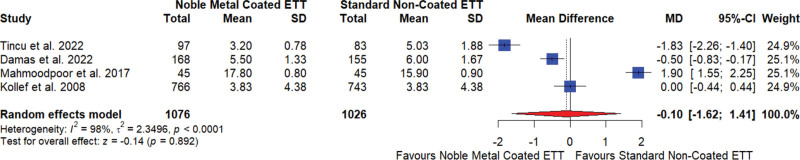
Duration of mechanical ventilation for noble element coated endotracheal tube (ETT) versus standard noncoated ETT. CI = confidence interval, MD = mean difference, SD = standard deviation.

The meta-analysis of 4 studies^[17,18,21,22]^ examining the impact of noble element–coated ETTs on the duration of mechanical ventilation in days found no significant difference compared to standard noncoated ETTs. The overall mean difference (MD) was −0.10, suggesting no notable effect on the duration of mechanical ventilation, with a 95% CI of −1.62 to 1.41 and a nonsignificant result (*P* = .892). The studies showed very high heterogeneity (I^2^ = 98%), indicating substantial variability in the results (Fig. 4).

The sensitivity analysis demonstrated that the overall effect size was stable across different study exclusions, with MDs ranging from −0.78 to 0.47 and consistently high heterogeneity (I2 ranging from 95% to 99%; Supplementary Data 02, Supplemental Digital Content, http://links.lww.com/MD/N601). The Egger test for publication bias indicated no significant bias (*P* = .5881).

#### 3.5.4. Duration of ICU stay (days)

**Figure 5.**
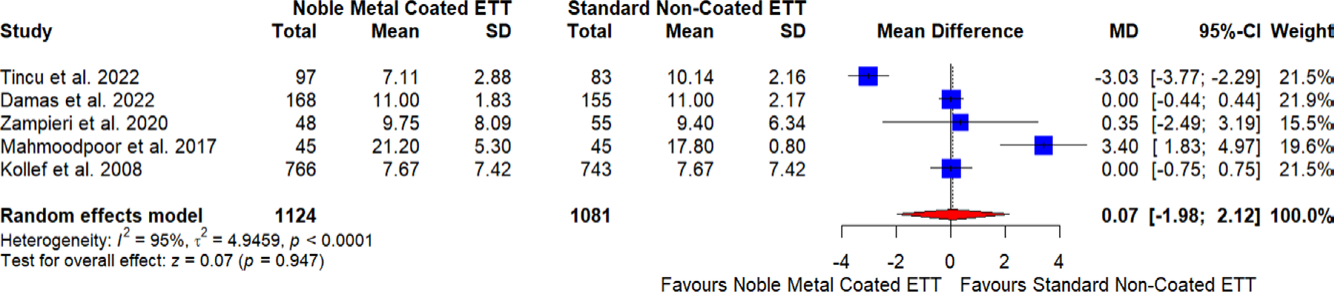
Duration of intensive care unit stays for noble element coated endotracheal tube (ETT) versus standard noncoated ETT. CI = confidence interval, MD = mean difference, SD = standard deviation.

The meta-analysis of 5 studies^[17,18,20–22]^ investigating the effect of noble element–coated ETTs on the duration of ICU stay in days showed no significant difference compared to standard noncoated ETTs. The overall MD was 0.07, indicating no significant effect on the duration of ICU stay, with a 95% CI of −1.98 to 2.12 and a nonsignificant result (*P* = .947). The studies showed very high heterogeneity (I^2^ = 95%), indicating substantial variability in the results (Fig. 5).

The sensitivity analysis revealed that the overall effect size was relatively stable across different study exclusions, with MDs ranging from −0.79 to 0.88 and consistently high heterogeneity (I^2^ ranging from 82% to 96%; Supplementary Data 02, Supplemental Digital Content, http://links.lww.com/MD/N601). The Egger test for publication bias showed no significant bias (*P* = .7843).

## 4. Discussion

The findings of this systematic review and meta-analysis demonstrated that noble element–coated ETTs reduced the incidence of VAP in comparison to standard noncoated ETT in the settings of emergency care. However, there was no significant effect on other outcomes such as mortality rate, duration of mechanical ventilation, and ICU stay. Similarly, in this review, 2 main types of ETT coatings were used: silver and alloy (gold, silver, and palladium), with a ratio of 4:3 studies. However, no significant differences were found in the overall findings.

In the literature, 2 previous reviews compared silver-coated and noncoated devices. Both reviews reported similar findings, indicating a reduction in VAP.^[12,13]^ Regarding the other outcomes, Li et al^[12]^ found no significant difference in mortality rate, while Tokmaji et al^[13]^ reported inconclusive findings for hospital mortality, duration of intubation, and length of hospital and ICU stay. However, both of these reviews included only the 2 RCTs, one of which^[21]^ was also part of our study. Apart from these reviews, a meta-analysis evaluated the efficacy of noble metal element catheters in reducing catheter-associated urinary tract infections in adult patients requiring short-term catheterization. It was concluded that silver alloy–coated catheters (RR, 0.63 [95% CI, 0.44–0.90]; *P* = .01; I^2^ = 72%) and noble metal alloy catheters (RR, 0.58 [95% CI, 0.41–0.81]; *P* = .001; I^2^ = 0%) significantly reduced the risk of bacteriuria.^[26]^

The effectiveness of noble element coatings in reducing VAP can be primarily attributed to their potent antimicrobial properties and ability to prevent biofilm formation. This antimicrobial effect is crucial in preventing the colonization of bacteria on the ETTs, which are a common site for infections leading to VAP.^[21,27,28]^ In addition, the noble element coatings significantly inhibit the formation of biofilms. These biofilms provide a stable environment for bacteria to thrive and protect them from antimicrobial agents.^[21,27,29]^ By preventing biofilm formation, noble element coatings reduce the risk of bacterial colonization and subsequent infection of VAP.

Despite their effectiveness in reducing VAP incidence, the lack of significant impact of ETT noble element coatings on mortality rate, duration of mechanical ventilation, and length of ICU stay can be attributed to several factors. VAP is only one of many complications that critically ill patients may encounter. Other factors, such as the severity of the underlying illness, the presence of comorbidities, and overall patient health, play crucial roles in determining the mortality rates, duration of mechanical ventilation, and ICU stay.^[12,30,31]^ In our included studies, patients were admitted for various severe conditions, including respiratory, neurological, cardiac, and surgical issues. Regarding comorbidities, 17.6% of participants in the intervention group had coexisting medical conditions. The overall health status, quantified by the mean Acute Physiology and Chronic Health Evaluation II score reported in 3 studies, was 20.6 for the intervention group, indicating a high-risk category.^[32]^ Therefore, it can be concluded that while the effectiveness of these coatings in reducing VAP incidence is evident, their ability to influence broader clinical outcomes is limited by the multifaceted nature of critical illness and the comprehensive care required for these patients. Consequently, these coatings should be incorporated into a holistic care plan that addresses all facets of patient management in emergency care settings.

In interpreting the findings of this review, several limitations should be acknowledged. First, the number of included studies was limited, with a total of 2418 participants, which may affect the generalizability of the results. In addition, the diagnostic criteria employed for VAP across the various studies were inconsistent, complicating the interpretation of the findings. While cost could be an important factor influencing the decision to use noble element–coated ETTs, the review did not assess the cost-effectiveness of these ETTs. Given these limitations, future studies should aim to expand the sample size to enhance the generalizability of the findings, develop and implement uniform diagnostic criteria to ensure consistency across studies, and include evaluations of cost-effectiveness to provide a more comprehensive understanding of the intervention. By addressing these limitations, future research can offer more robust and applicable insights into patient management in emergency care settings.

## 5. Conclusion

Noble element–coated ETTs significantly reduce the incidence of VAP compared to standard noncoated ETTs in emergency care settings. However, these coatings do not appear to affect other key outcomes, such as mortality rates, duration of mechanical ventilation, or length of ICU stay, likely due to the complex nature of critical illness and the need for comprehensive patient care. Therefore, while coated ETTs can be a valuable component of patient management, they should be integrated into a broader, holistic care plan. To strengthen future research, studies should aim to increase sample sizes to improve generalizability, establish uniform VAP diagnostic criteria for consistency across studies, and include cost-effectiveness analyses to provide a more complete assessment of the intervention.

## Author contributions

**Conceptualization:** Nabeel Ashiq, Fouzia Munir, Safeer Khan.

**Data curation:** Nabeel Ashiq, Safeer Khan.

**Formal analysis:** Nabeel Ashiq, Fouzia Munir, Safeer Khan.

**Methodology:** Nabeel Ashiq, Fouzia Munir, Adil Yousaf, Malik Hassan Mahmood.

**Writing – review & editing:** Nabeel Ashiq, Safeer Khan.

**Supervision:** Fouzia Munir, Malik Hassan Mahmood.

**Writing – original draft:** Fouzia Munir, Adil Yousaf.

**Investigation:** Adil Yousaf.

**Software:** Adil Yousaf, Malik Hassan Mahmood.

## Supplementary Material



## References

[1] Koenig SM Truwit JD. Ventilator-associated pneumonia: diagnosis, treatment, and prevention. Clin Microbiol Rev. 2006;19:637–57. 17041138 10.1128/CMR.00051-05 PMC1592694

[2] Kalanuria AA Ziai W Mirski M. Ventilator-associated pneumonia in the ICU. Crit Care. 2014;18:1–8. 10.1186/cc13775 PMC4056625 25029020

[3] Papazian L Klompas M Luyt CE. Ventilator-associated pneumonia in adults: a narrative review. Intensive Care Med. 2020;46:888–906. 32157357 10.1007/s00134-020-05980-0 PMC7095206

[4] Wagh H Acharya D. Ventilator associated pneumonia--an overview. Br J Med Pract. 2009;2:16–9.

[5] Scott RD. The direct medical costs of healthcare-associated infections in US hospitals and the benefits of prevention. Division of Healthcare Quality Promotion National Center for Preparedness, Detection, and Control of Infectious Diseases Coordinating Center for Infectious Diseases Centers for Disease Control and Prevention, Atlanta. 2009.

[6] Kalil AC Metersky ML Klompas M. Management of adults with hospital-acquired and ventilator-associated pneumonia: 2016 clinical practice guidelines by the Infectious Diseases Society of America and the American Thoracic Society. Clin Infect Dis. 2016;63:e61–e111. 27418577 10.1093/cid/ciw353 PMC4981759

[7] Torres A Niederman MS Chastre J. International ERS/ESICM/ESCMID/ALAT guidelines for the management of hospital-acquired pneumonia and ventilator-associated pneumonia: guidelines for the management of hospital-acquired pneumonia (HAP)/ventilator-associated pneumonia (VAP) of the European Respiratory Society (ERS), European Society of Intensive Care Medicine (ESICM), European Society of Clinical Microbiology and Infectious Diseases (ESCMID) and Asociación Latinoamericana del Tórax (ALAT). Eur Respir J. 2017;50:1700582. 28890434 10.1183/13993003.00582-2017

[8] Klompas M. Oropharyngeal decontamination with antiseptics to prevent ventilator-associated pneumonia: rethinking the benefits of chlorhexidine. Semin Respir Crit Care Med. 2017;38:381–90. 28578560 10.1055/s-0037-1602584

[9] Diaconu O Siriopol I Poloșanu LI Grigoraș I. Endotracheal tube biofilm and its impact on the pathogenesis of ventilator-associated pneumonia. J Crit Care Med (Targu Mures). 2018;4:50–5. 30581995 10.2478/jccm-2018-0011 PMC6294989

[10] Fernandez JF Levine SM Restrepo MI. Technologic advances in endotracheal tubes for prevention of ventilator-associated pneumonia. Chest. 2012;142:231–8. 22796845 10.1378/chest.11-2420 PMC3418858

[11] Alves D Grainha T Pereira MO Lopes SP. Antimicrobial materials for endotracheal tubes: a review on the last two decades of technological progress. Acta Biomater. 2023;158:32–55. 36632877 10.1016/j.actbio.2023.01.001

[12] Li X Yuan Q Wang L Du L Deng L. Silver-coated endotracheal tube versus non-coated endotracheal tube for preventing ventilator-associated pneumonia among adults: a systematic review of randomized controlled trials. J Evid Based Med. 2012;5:25–30. 23528117 10.1111/j.1756-5391.2012.01165.x

[13] Tokmaji G Vermeulen H Müller MC Kwakman PH Schultz MJ Zaat SA. Silver-coated endotracheal tubes for prevention of ventilator-associated pneumonia in critically ill patients. Cochrane Database Syst Rev. 2015;2015:CD009201. 26266942 10.1002/14651858.CD009201.pub2 PMC6517140

[14] Liberati A Altman DG Tetzlaff J. The PRISMA statement for reporting systematic reviews and meta-analyses of studies that evaluate health care interventions: explanation and elaboration. Ann Intern Med. 2009;151:W65–94. 19622512 10.7326/0003-4819-151-4-200908180-00136

[15] Sterne JAC Savović J Page MJ. RoB 2: a revised tool for assessing risk of bias in randomised trials. BMJ. 2019;366:l4898. 31462531 10.1136/bmj.l4898

[16] Higgins JP Thompson SG Deeks JJ Altman DG. Measuring inconsistency in meta-analyses. BMJ. 2003;327:557–60. 12958120 10.1136/bmj.327.7414.557 PMC192859

[17] Tincu RC Cobilinschi C Tincu IF Macovei RA. Efficacy of noble metal–alloy endotracheal tubes in ventilator-associated pneumonia prevention: a randomized clinical trial. Balkan Med J. 2022;39:167–71. 35332771 10.4274/balkanmedj.galenos.2021.2021-7-86 PMC9136541

[18] Damas P Legrain C Lambermont B. Prevention of ventilator-associated pneumonia by noble metal coating of endotracheal tubes: a multi-center, randomized, double-blind study. Ann Intensive Care. 2022;12:1. 34981245 10.1186/s13613-021-00961-y PMC8723906

[19] Mahmodiyeh B Kamali A Zarinfar N Joushani MM. The effect of silver-coated endotracheal tube on the incidence of ventilator-induced pneumonia in intubated patients admitted to the intensive care unit (ICU). Syst Rev Pharm. 2021;12:254–8.

[20] Zampieri FG de Oliveira NE Nassar AP.; BRICNet. Bundle of coated devices to reduce nosocomial infections in the intensive care unit. CRITIC pilot randomized controlled trial. Ann Am Thorac Soc. 2020;17:1257–63. 32526149 10.1513/AnnalsATS.202003-206OC

[21] Kollef MH Afessa B Anzueto A.; NASCENT Investigation Group. Silver-coated endotracheal tubes and incidence of ventilator-associated pneumonia: the NASCENT randomized trial. JAMA. 2008;300:805–13. 18714060 10.1001/jama.300.7.805

[22] Mahmoodpoor A Sanaie S Parthvi R. A clinical trial of silver-coated and tapered cuff plus supraglottic suctioning endotracheal tubes in preventing ventilator-associated pneumonia. J Crit Care. 2020;56:171–6. 31935605 10.1016/j.jcrc.2019.12.024

[23] Afessa B. Silver-coated endotracheal tubes and incidence of ventilator-associated pneumonia. The NASCENT study: a randomized trial. 2010;137:1015–21. 10.1001/jama.300.7.805 18714060

[24] Zilberberg MD Shorr AF. Ventilator-associated pneumonia: the clinical pulmonary infection score as a surrogate for diagnostics and outcome. Clin Infect Dis. 2010;51:S131–5. 20597663 10.1086/653062

[25] Mahmodiyeh B Kamali A Zarinfar N Joushani MM. The effect of silver-coated endotracheal tube on the incidence of ventilator-induced pneumonia in intubated patients admitted to the Intensive Care Unit (ICU). Syst Rev Pharm. 2021;12:254–62.

[26] Sun Y Ren P Long X. Role of noble metal-coated catheters for short-term urinary catheterization of adults: a meta-analysis. PLoS One. 2020;15:e0233215. 32520937 10.1371/journal.pone.0233215 PMC7286480

[27] Berra L De Marchi L Yu ZX Laquerriere P Baccarelli A Kolobow T. Endotracheal tubes coated with antiseptics decrease bacterial colonization of the ventilator circuits, lungs, and endotracheal tube. Anesthesiology. 2004;100:1446–56. 15166564 10.1097/00000542-200406000-00017

[28] Suska F Svensson S Johansson A. In vivo evaluation of noble metal coatings. J Biomed Mater Res B Appl Biomater. 2010;92:86–94. 19701914 10.1002/jbm.b.31492

[29] Vaishampayan A De Jong A Wight DJ Kok J Grohmann E. A novel antimicrobial coating represses biofilm and virulence-related genes in methicillin-resistant Staphylococcus aureus. Front Microbiol. 2018;9:315696. 10.3389/fmicb.2018.00221 PMC5818464 29497410

[30] Combes A Luyt CE Fagon JY Wolff M Trouillet JL Chastre J. Impact of piperacillin resistance on the outcome of Pseudomonas ventilator-associated pneumonia. Intensive Care Med. 2006;32:1970–8. 16957901 10.1007/s00134-006-0355-7

[31] Tseng CC Liu SF Wang CC. Impact of clinical severity index, infective pathogens, and initial empiric antibiotic use on hospital mortality in patients with ventilator-associated pneumonia. Am J Infect Control. 2012;40:648–52. 22243991 10.1016/j.ajic.2011.08.017

[32] Knaus WA Draper EA Wagner DP Zimmerman JE. APACHE II: a severity of disease classification system. Crit Care Med. 1985;13:818–29. 3928249

