# Supplementary material for: Noble element coatings on endotracheal tubes for ventilator-associated pneumonia prevention: A systematic review and meta-analysis of randomized controlled trials in emergency care settings
Source: Medicine (Baltimore). 2024 Sep 20;103(38):e39750. doi: 10.1097/MD.0000000000039750 (PMC11419469; doi:10.1097/MD.0000000000039750)
Supplement: Supplementary file 1 [file medi-103-e39750-s001.docx]

**Search Strategy**

| **Keywords** | **Database** | **No. of retrieved results** |
| --- | --- | --- |
| ("noble"[All Fields] OR "nobles"[All Fields]) AND ("element s"[All Fields] OR "elements"[MeSH Terms] OR "elements"[All Fields] OR "element"[All Fields]) AND ("pneumonia"[MeSH Terms] OR "pneumonia"[All Fields] OR "pneumonias"[All Fields] OR "pneumoniae"[All Fields] OR "pneumoniae s"[All Fields]) AND ("tube s"[All Fields] OR "tubed"[All Fields] OR "tubes"[All Fields] OR "tubing"[All Fields] OR "tubings"[All Fields]) | PubMed | 1 |
| ("platinum"[MeSH Terms] OR "platinum"[All Fields] OR "platinums"[All Fields]) AND ("pneumonia"[MeSH Terms] OR "pneumonia"[All Fields] OR "pneumonias"[All Fields] OR "pneumoniae"[All Fields] OR "pneumoniae s"[All Fields]) AND ("tube s"[All Fields] OR "tubed"[All Fields] OR "tubes"[All Fields] OR "tubing"[All Fields] OR "tubings"[All Fields]) | PubMed | 2 |
| ("pneumonia"[MeSH Terms] OR "pneumonia"[All Fields] OR "pneumonias"[All Fields] OR "pneumoniae"[All Fields] OR "pneumoniae s"[All Fields]) AND (("endotracheal"[All Fields] OR "endotracheally"[All Fields]) AND ("tube s"[All Fields] OR "tubed"[All Fields] OR "tubes"[All Fields] OR "tubing"[All Fields] OR "tubings"[All Fields])) | PubMed | 352 |
| ("gold"[MeSH Terms] OR "gold"[All Fields]) AND ("pneumonia"[MeSH Terms] OR "pneumonia"[All Fields] OR "pneumonias"[All Fields] OR "pneumoniae"[All Fields] OR "pneumoniae s"[All Fields]) AND (("endotracheal"[All Fields] OR "endotracheally"[All Fields]) AND ("tube s"[All Fields] OR "tubed"[All Fields] OR "tubes"[All Fields] OR "tubing"[All Fields] OR "tubings"[All Fields])) | PubMed | 3 |
| ("silver"[MeSH Terms] OR "silver"[All Fields] OR "silvers"[All Fields] OR "silvered"[All Fields]) AND ("pneumonia"[MeSH Terms] OR "pneumonia"[All Fields] OR "pneumonias"[All Fields] OR "pneumoniae"[All Fields] OR "pneumoniae s"[All Fields]) AND (("endotracheal"[All Fields] OR "endotracheally"[All Fields]) AND ("tube s"[All Fields] OR "tubed"[All Fields] OR "tubes"[All Fields] OR "tubing"[All Fields] OR "tubings"[All Fields])) | PubMed | 38 |
| allintitle: Pneumonia AND Endotracheal tubes | Google Scholar | 52 |
| Title, abstract, keywords: Pneumonia AND Endotracheal tubes | Science Direct | 194 |
| Endotracheal tubes AND Pneumonia):ab" in Cochrane Reviews, Trials, Editorials (Word variations have been searched) | Cochrane Library | 360 |
| pneumonia endotracheal tubes ( TITLE ( pneumonia ) AND TITLE ( endotracheal AND tubes ) ) | Scopus | 109 |
