# Supplementary material for: Noble element coatings on endotracheal tubes for ventilator-associated pneumonia prevention: A systematic review and meta-analysis of randomized controlled trials in emergency care settings
Source: Medicine (Baltimore). 2024 Sep 20;103(38):e39750. doi: 10.1097/MD.0000000000039750 (PMC11419469; doi:10.1097/MD.0000000000039750)
Supplement: Supplementary file 2 [file medi-103-e39750-s002.docx]

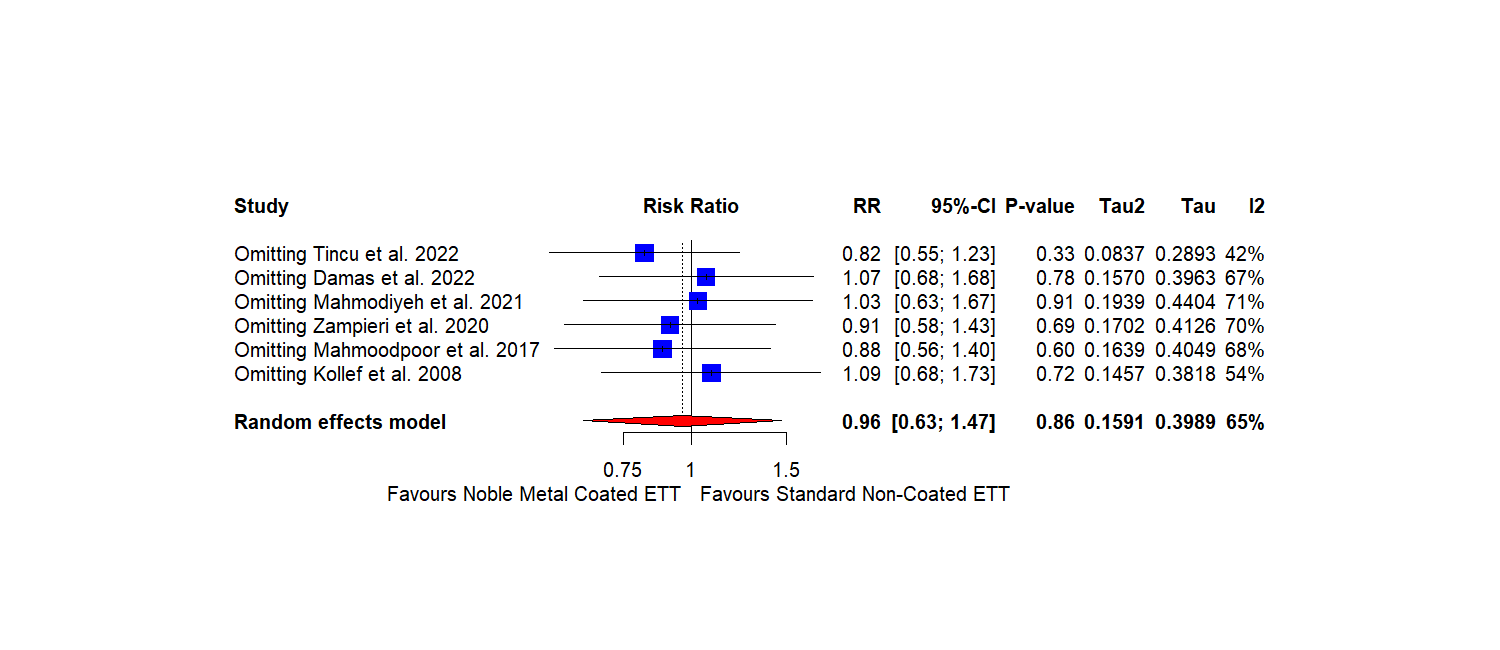


Figure. Sensitivity Analysis (VAP)


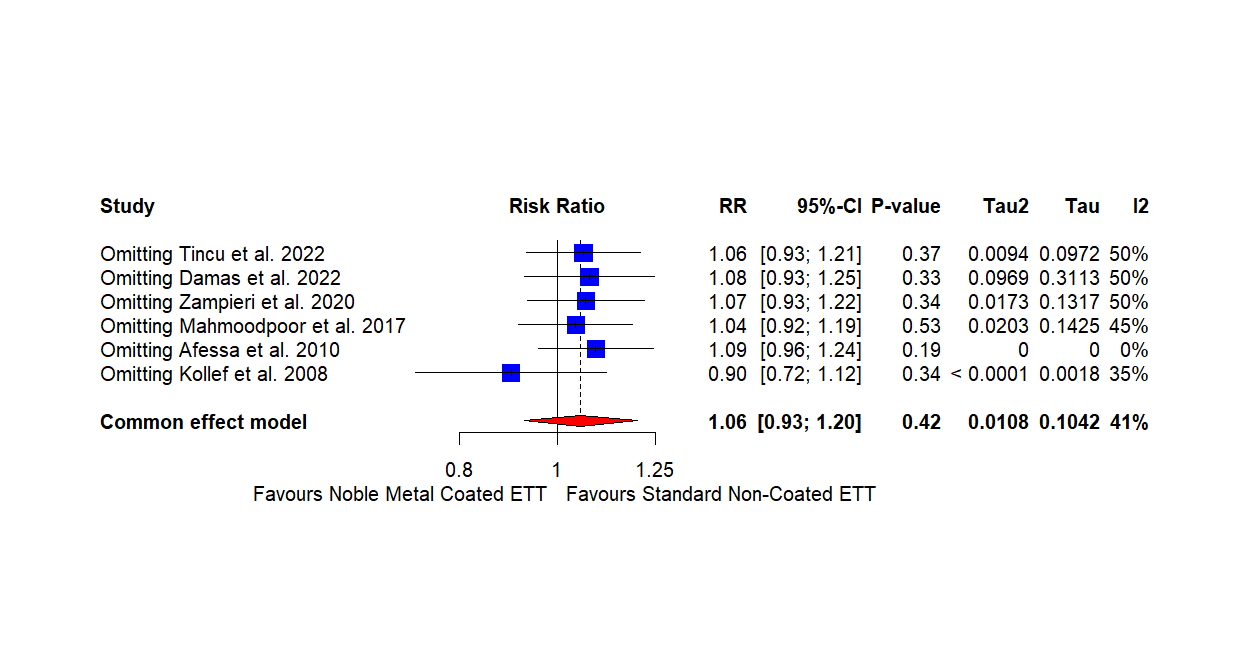
Figure. Sensitivity Analysis (Mortality Rate)


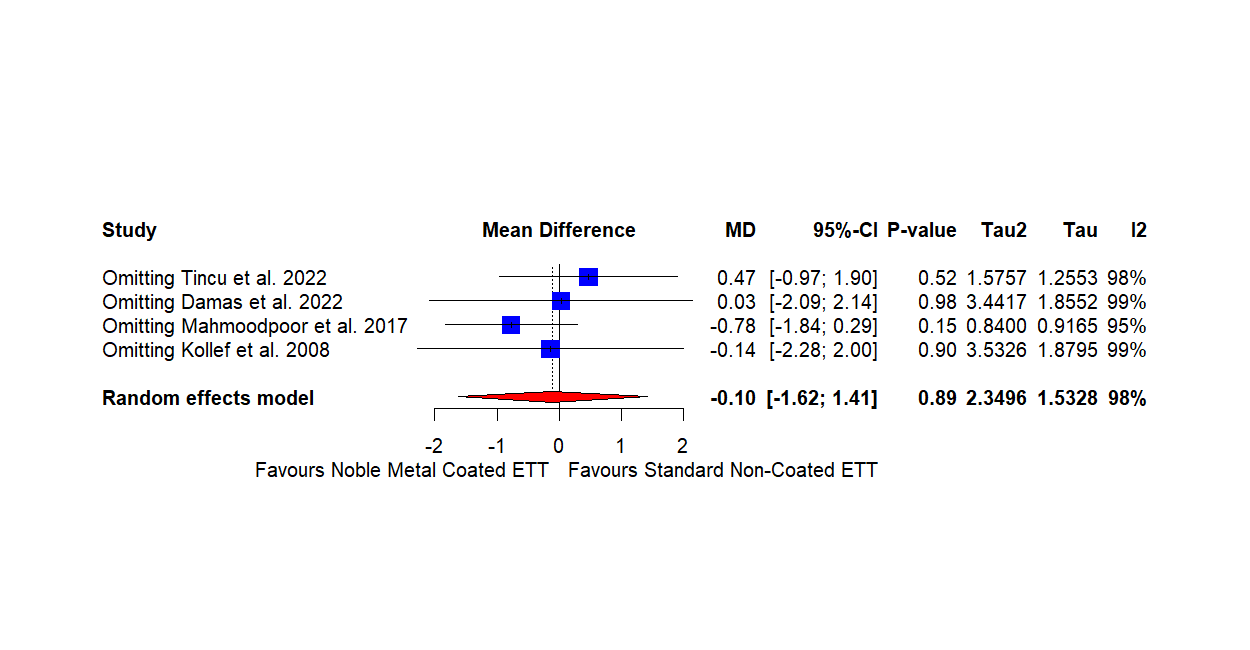
Figure. Sensitivity Analysis (Duration of Mechanical Ventilation)


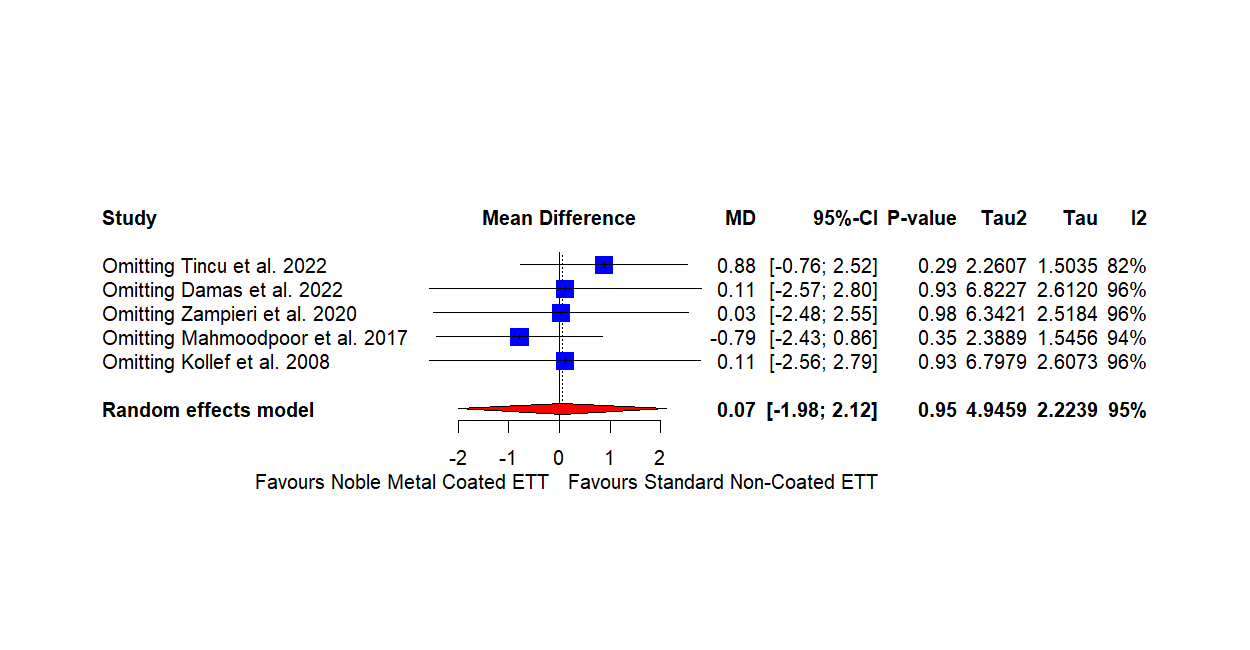
Figure. Sensitivity Analysis (ICU Stay)
